# Unequal smiles: consequences of untreated dental caries in citizens living in vulnerable circumstances in the Netherlands: an exploratory pilot study

**DOI:** 10.2340/aos.v83.42028

**Published:** 2024-10-03

**Authors:** Sterre J. Gitz, Geert J. M. G. van der Heijden, Catherine M.C. Volgenant

**Affiliations:** a Department of Cariology, Academic Centre of Dentistry Amsterdam (ACTA), University of Amsterdam and Vrije Universiteit Amsterdam, Amsterdam, the Netherlands; b Department of Oral Public Health, Academic Centre of Dentistry Amsterdam (ACTA), University of Amsterdam and Vrije Universiteit Amsterdam, Amsterdam, the Netherlands; c Department of Preventive Dentistry, Academic Centre of Dentistry Amsterdam (ACTA), University of Amsterdam and Vrije Universiteit Amsterdam, Amsterdam, the Netherlands

**Keywords:** Untreated dental caries, dental caries, odontogenic infection, oral health-related quality of life, social class, poverty, oral healthcare services

## Abstract

The often poor oral health status of socioeconomically vulnerable adults is widely recognised. Nevertheless, research on it is scarce. To address this gap, this exploratory pilot study aimed to report on the prevalence of untreated caries and its clinical odontogenic consequences, as well as the associated Oral Health Related Quality of Life (OHRQoL) in a marginalised adult Dutch population.

The Dutch department of *Médecins du Monde* (Doctors of the World) included socioeconomically vulnerable (low socioeconomic position [SEP]) adults in the Netherlands through community organisations. The validated Deprivation in Primary Care Questionnaire (DiPCare-Q) was translated in Dutch and used to characterise the SEP of the population. To document untreated caries and severe odontogenic consequences, the DMFT (Decayed, Missing, Filled Permanent Teeth) and PUFA (Pulpal, Fistula, Ulceration, Abscess) index were used. The validated Dutch Oral Health Impact Profile questionnaire (OHIP-14) was used to document the impact of these issues on OHRQoL. Data analysis was conducted in SPSS® (Statistical Package for the Social Sciences) statistics (Kruskal-Wallis, Mann-Whitney-U-test) and STATA software.

Data from 59 adult participants were analysed. The prevalence of untreated caries (DT ≥ 1) was 65.5%, 57.9% of which experienced severe odontogenic consequences (DT+PUFA). The prevalence of severe odontogenic consequences (PUFA ≥ 1) was 45.5%. The mean OHIP-14 score of 17.7 ± 13.4 (25th–75th percentile: 6–26) illustrated that untreated caries may have impact on OHRQoL. Individuals who experienced any severe odontogenic consequences from untreated caries reported significantly higher OHIP-14 scores (mean ± s.d.: 21.8 ± 14.8 vs.11.1 ± 7.2).

The untreated caries and severe odontogenic consequences seen in a large number of the socioeconomically vulnerable adult participants seemed to have an impact on OHRQoL. These findings emphasise the urgency of including these adults in the professional oral health system for treatment and prevention, and may emphasise the necessity of improving the socioeconomic circumstances of this population. Further exploration of the exact barriers and facilitators to oral healthcare access for socioeconomically vulnerable adults is necessary.

## Introduction

Oral diseases, such as untreated caries still affect almost half of the global population [1, 2]. If left untreated, caries can cause pain and impair functioning, which can lead to problems with diet, speech and confidence, resulting in severe consequences for physical, social and psychological wellbeing and, hence, decreased Oral Health Related Quality of Life (OHRQoL) [1, 3]. Marginalised groups in the Netherlands experience more oral diseases than the general population, while access to and the affordability of professional oral healthcare are limited [1, 3–12].

The mechanisms of socioeconomic inequalities in oral health are complex and therefore not yet fully understood [4, 13]. Material factors such as financial constraints are often indicated as obvious explanations for socioeconomic inequalities in oral health [14, 15]. However, other factors, such as social, cultural, behavioural and psychosocial factors, are also associated with these inequalities [4, 16]. Accordingly, addressing oral health inequality is a significant challenge for health policymakers worldwide [5, 17].

A factor that contributes to this challenge is the lack of knowledge about the oral health status of marginalised groups. This issue has arisen because of the difficulties involved in accessing and engaging these groups for research purposes. As a result, they are often omitted from research and referred to as ‘hidden’ or ‘hard-to-reach’ populations [18–21]. They are considered hard to reach because of cultural barriers, mistrust towards researchers, fear for research and various practical factors. For example, they are often absent from the recruitment sites that are typically used by oral health researchers [20, 21]. Participants for oral health research are often recruited from dental practices, health centres, and government institutions – locations where the marginalised are rarely seen [15, 22].

To date, few studies in the Netherlands have tried to access and engage these marginalised communities. Some studies have reported on the caries prevalence and experience of disadvantaged groups who did manage to visit the dental office [11, 23]. However, they do not describe the consequences of untreated caries in terms of odontogenic infections or report on OHRQoL. This gap in knowledge hinders the development of effective interventions to address oral health disparities. The importance of researching this gap is growing given the increasing poverty levels in the Netherlands in recent years [24, 25]. A rising number of individuals will be living in vulnerable circumstances, which will consequently jeopardise their overall health, including oral health. This stresses the urgency of addressing oral health inequalities.

To address this gap, this study used the Pulpal, Fistula, Ulceration, Abscess (PUFA) index to report clinical odontogenic consequences of a potentially high prevalence of untreated caries. The Deprivation in Primary Care Questionnaire (DiPCare-Q) questionnaire was used to accurately capture and describe the social context of this population. This approach has not yet been applied to such a population in the Netherlands within oral healthcare research [26, 27]. The aim of this cross-sectional explorative pilot study was to report the prevalence of untreated caries and its clinical odontogenic consequences, as well as the associated OHRQoL, in a Dutch adult population with low socioeconomic position (SEP) and limited access to routine professional oral healthcare [26, 27].

## Methods

### Design and setting

Data collection was conducted using a cross-sectional design. The study setting included four community organisations in five municipalities in the Netherlands. These were: Waalre and Almere: Foodbank; Goes: the Salvation army; The Hague: Het Wereld Huis, a support organisation for undocumented immigrants; Rotterdam: Centrum voor Dienstverlening (CVD), a center for social care, social services and assistance in crisis situations. These locations were assumed to serve socioeconomically vulnerable (low SEP) adults. Rotterdam and The Hague (province South Holland) and Almere (province Flevoland) are urban locations. Waalre (province Noord-Brabant) and Goes (province Zeeland) are intermediate and rural locations, respectively (Eurostat urban-rural typology) [28]. The Dutch department of *Médecins du Monde* (Doctors of the World) acted as an intermediary in contacting these community organisations and including the participants. They also intermediated in organising free dental treatment for the participants in either a mobile dental clinic at the location of the participating organisation (provided by NoviaCura B.V.) or the dental clinic at the Erasmus Medical Centre in Rotterdam for participants included from CVD. The protocol for this study was approved by the ACTA Internal Review Board in August 2021, registration number 2021-14626. This study was reported according to the *Strengthening the Reporting of Observational Studies in Epidemiology (STROBE)* statement [29].

### Participant inclusion

Participants were included through convenience sampling from four community organisations that have access to marginalised groups. These organisations were included by Doctors of the World through convenience sampling based on logistical and financial considerations for the foundation. Participants were eligible if they were: adults (21+ years old), used the service(s) of the community organisations, responded to the announcement from Doctors of the World regarding the availability of free dental care by a qualified dentist, and were seen at the treatment location (i.e., their community organisation) for a first dental visit. All participants provided written informed consent to participate prior to data collection.

### Data collection

Two validated questionnaires were used: the DiPCare-Q, translated to Dutch without subsequent validation, and the abbreviated Oral Health Impact Profile (OHIP-14) [27, 30]. Two bilingual researchers independently reviewed the translation of the DiPCare-Q to include all nuances and afterwards face validity has been determined. Two individuals (one volunteer and one researcher) either read the questionnaires, which were available in English and Dutch, to the participants or provided clarification if needed prior to the intraoral examination. Additionally, a form was used to collect personal data on sex, age, community organisations, tooth brushing frequency, date of last visit to a dentist, level of education, employment (dichotomous), immigration background (dichotomous), and reasons for forgoing routine professional oral healthcare. The presence of an immigration background was defined as being a first or second-generation immigrant in the Netherlands.

To depict the study population’s SEP, the DiPCare-Q was used in addition to level of education. The DiPCare-Q records an individual’s level of deprivation over the previous 1, 3 and 12 months. The questions are designed to align with Townsend’s definition of deprivation and are predicated on the understanding that social determinants are significant factors that can negatively influence health and behaviour [31]. It is composed of 16 dichotomous questions weighted at one point per question and which belong to three dimensions (material, social, health) [27]. The total DiPCare-Q *sum score* can range from 0 (no deprivation) to 16 (highest deprivation) points, and the sum scores per dimension from 0 to 8 points, material dimension; 0 to 5 points, social dimension; and 0 to 3 points, health dimension. The DiPCare-Q *overall index* was computed in Stata/SE using the equation: Overall Index = 0.810*[material deprivation] + 0.455*[social deprivation] + 0.711*[health deprivation]. The overall index can range from 0 (no deprivation) to 5 (highest deprivation) points [27]. To establish the impact of oral conditions on self-reported OHRQoL over the last month, the OHIP-14 was used. The OHIP-14 is derived from the larger OHIP-49 questionnaire. It is composed of 14 questions that still cover the same seven dimensions: functional limitations, physical pain, psychological discomfort, physical disability, psychological disability, social disability and handicaps. Possible answers are distributed in a Likert scale (4 points = ‘very often’, 3 points = ‘often’, 2 points = ‘sometimes’, 1 point = ‘rarely’, 0 = ‘never’). These dimensions represent the frequency in which participants experience the impact of the oral condition on their lives. The total OHIP-14 sum score ranges from 0 (good OHRQoL) to 56 (poor OHRQoL) [30].

### Intraoral data collection

Three experienced and beforehand instructed dentists conducted the intraoral examinations using a mirror, probe, light source and air water spray syringe to score the DMFT and PUFA indices. Third molars were excluded from both indices [23, 26]. A DT was recorded for a clinical ICDAS (International Caries Detection and Assessment System) score of 3 or higher, indicating ‘clinically visible tissue loss of the enamel and/or dentine surface or a visible dark shadow in the underlying dentine’ was present [32]. The DMFT index determines the sum of teeth affected by caries in terms of decay (DT), missing because of caries (MT) and filled because of caries (FT) permanent teeth, collectively referred to as ‘caries experience’. The PUFA index determined the severe odontogenic consequences resulting from untreated caries. The components of this index represent:

- P: Pulpal involvement for severely decayed teeth with visible open pulp chambers and/or radix remains in the jaw;
- U: Ulceration visible in the surrounding soft tissue because of sharp teeth fragments;
- F: Fistula present in the surrounding soft tissue related to the tooth with pulpal involvement, or
- A: Abscess present in the surrounding soft tissue related to the tooth with pulpal involvement [26].

Teeth can only be assigned to one component of this index. If the extent of the odontogenic infection raises uncertainty, a P (for Pulpal involvement) is given. Similar to the DMFT index, the PUFA index represents the number of PUFA affected teeth, therefore it can range from 0 to 28. The PUFA index is not recorded for edentulous participants. Missing teeth are not recorded in the PUFA index.

### Design of data analysis

Data analysis was conducted in IBM® SPSS® (Statistical Package for the Social Sciences) statistics, version 28.0 (IBM Inc., SPSS 28.0, NY, USA) and in Stata/SE version 16 (StataCorp LLC, College Station, TX, USA). A non-Gaussian distribution was assumed for all variables.

#### Missing data

Missing data were handled by excluding cases with missing data for one or more of the analysed variables.

#### Population descriptives

The DiPCare-Q results were reported as the median *sum score* for the total population and per dimension. Additionally, the percentage prevalence per dimension and item was reported alongside the *overall index*, as these results were considered highly indicative for characterising the population. Participants were classified as deprived in a DiPCare-Q dimension if they scored ‘deprived’ on at least one item in that dimension (i.e., a ‘yes’ in the material and health dimensions, or a ‘no’ in the social dimension).

The prevalence of the teeth with untreated caries (DT) and teeth with severe odontogenic consequences (PUFA) in the study population were described as the percentage of the population with a minimum score of one. Additionally, the prevalence of severe odontogenic consequences in participants with untreated caries (DT+PUFA) was reported. The caries experience (DMFT), untreated caries experience (DT), the experience of severe odontogenic consequences (PUFA) of the study population and in participants with untreated caries (DT+PUFA) were described as mean scores with standard deviations and 95% confidence intervals (CIs). The ‘Untreated Caries, PUFA Ratio’ provided information on tooth level as the percentage of teeth with untreated caries that developed an odontogenic infection and was calculated with the formula [PUFA/DT*100] [26]. OHIP-14 was described as mean score with standard deviation (s.d.) and 25th and 27th percentiles.

#### Comparative analyses

For details on variable recoding, we refer to supplementary material: ‘variable recoding’. The experience of untreated caries (DT), severe odontogenic consequences (PUFA) and OHRQoL (OHIP-14) were compared between subpopulations based on demographic factors (level of education, brushing frequency, last dentist visit). OHRQoL was additionally compared by clinical variables (untreated caries, severe odontogenic consequences). The Mann-Whitney U test and Kruskal-Wallis test were used to test for statistical significant differences (*p* < 0.05).

## Results

### Participants

Of the 60 participants shortlisted by Doctors of the World through the participating community organisations, one was excluded for not meeting the age criteria. Consequently, data of 59 individuals were analysed.

### Missing data

The common feature observed among study participants with missing data was the municipality in which they received treatment and, consequently, the type of community organisation that engaged them; the Foodbank in Almere. The missing data almost entirely concerned non-intraoral data and was the result of time constraints throughout the treatment day.

### Population descriptives

The study population’s sociodemographic details and reasons for forgoing professional oral healthcare are presented in Table 1. The mean age in the study population was 42.5 years (s.d. = 12.9; range: 21–66; 95% CI [39.0, 45.9]). Forty participants (74.5%) last visited the dentist 2 years ago or more, of which 16 (40%) last visited more than 5 years ago. Three participants were edentulous (5.2%). The prevalence of sociodemographic variables in the study population compared with the general Dutch population are presented in the Supplementary material, Table S1.

**Table 1:** Sociodemographic details and reasons for forgone professional oral healthcare of the study population (*N* = 59).

Characteristics	Frequency *n*	%
Sex, male (missing = 1)	31	53.4
Community organisation-type		
Food bank	25	42.4
CVD	20	33.9
Undocumented immigrant organisation	8	13.6
Salvation army	6	10.2
Immigration background (missing = 1)	40	69.0
Level of education (missing = 7)		
Low	22	42.3
Middle	23	44.2
High	7	13.5
Currently unemployed (missing = 5)	47	87.0
Last dentist visit ≥ 2 years ago (missing = 4)	40	74.5
Brushing < 2 time a day (missing = 4)	24	43.6
Reason for forgone professional oral healthcare (missing = 6)		
Financial reasons	36	68.0
Fear and/or shame	11	20.8
Fear of having undocumented status discovered	7	13.2
No necessity	5	9.5
Personal circumstances	3	5.7
Difficulty in access	1	1.9
No reason	3	5.7

#### Socioeconomic characteristics

The overall DiPCare-Q index of the study population was 3.1 (s.d. = 1.1; range: 0–5, *n* = 54). The median DiPCare-Q sum score of the study population was 8 (interquartile range [IQR] 6–11; range: 1–15). The separate DiPCare-Q items illustrate that 51 (94.4%) participants suffered on a material level, all 54 (100%) participants suffered on a social level and to a lesser extent 32 (59.3%) participants suffered on a health level. While 50 (92.6%) participants were unable to afford holidays or leisure and recreational activities, 37 (68.5%) participants experienced the need to borrow money for daily expenses. Furthermore, 35 (64.8%) participants were unable to afford clothing and 34 (63.0%) participants experienced difficulty paying household bills. Although health issues were less apparent, the data demonstrated that 25 (46.3%) participants suffered from mental health issues. A full overview of results per dimension and item can be found in Table S2 in the Supplementary material.

#### Prevalence and odontogenic consequences of untreated caries and the OHRQoL

A high prevalence of untreated caries (DT) and severe odontogenic consequences (PUFA) were observed in the study population for 38 (65.5%) and 25 (45.5%) participants, respectively. Of those who experienced untreated caries, 22 (57.9%) participants experienced severe odontogenic consequence (DT+PUFA). Severe odontogenic consequences predominantly concerned Pulpal (P) involvements (24 participants, 43.6%). Only two (3.6%) participants experienced Fistulas (F), and one (1.8%) participant experienced an Abscess (A) in addition to the pulpal involvement on that tooth. No participants experienced Ulcerations (U). On tooth level the ‘Untreated Caries, PUFA Ratio’ shows that 36.6% of all teeth with untreated caries (DT) had developed into severe odontogenic consequences (PUFA). The mean OHIP-14 score for the entire study population was 17.7 (s.d. = 13.4; 25th–75th percentile: 6–26; *n* = 54). Painful aching and discomfort while eating were the most prevalent problems among participants (OHIP-14 item 3 and 4).

### Comparative analyses

Caries experience (DMFT score) of the study population was 12.3 (s.d. = 8.2; 95% CI [10.1, 14.5]; missing = 1) and the experience of untreated caries with severe odontogenic consequences (DT+PUFA) was 1.7 (s.d. = 2.6; 95% CI [0.8, 2.6]; *n* = 38). Mean DT, PUFA and OHIP-14 scores of the entire study population and subpopulations based on relevant demographic and clinical variables can be found in Table 2. The mean OHIP-14 scores of participants who experienced severe odontogenic consequences were significantly higher, indicating a poorer OHRQoL than participants who did not experience severe odontogenic consequences (*p* < 0.05; Table 2). This did not apply to the presence of untreated caries alone (*p* > 0.05). As a result of the homogeneity of the population in terms of SEP, no association was found for the overall DiPCare-Q index or sum scores for separate DiPCare-Q dimensions with items, and PUFA score, DT score, brushing frequency, last dentist visit or OHIP-14 score (*p* > 0.05).

**Table 2:** Mean DT, PUFA and OHIP-14 scores of the entire study population and subpopulations based on demographic and clinical variables.

Population	DT + s.d.	95% CI	PUFA + s.d.	95% CI	OHIP-14 + s.d.	95% CI
Entire study population	3.3 ± 4.7^#^	2.1 – 4.5	1.3 ± 2.3^##^	0.7 – 1.9	17.7 ± 13.4^###^	-
Subpopulations						
Level of education (missing = 8)						
Low (*n* = 22)	4.0 ± 5.5	1.5 – 6.5	1.5 ± 2.5	0.3 – 2.6	-	-
Middle (*n* = 23)	2.8 ± 4.6	0.8 – 4.8	1.6 ± 2.7	0.5 – 2.8	-	-
High (*n* = 6)	2.5 ± 3.7	−1.4 – 6.4	0.7 ± 1.0	−0.4 – 1.8	-	-
Brushing frequency (missing = 5)						
<2 times a day (*n* = 23)	4.2 ± 6.3	1.5 – 6.9	1.8 ± 2.8	0.5 – 3.1	18.5 ± 13.7	12.7 – 24.2
≥2 times a day (*n* = 31)	2.6 ± 3.2	1.4 – 3.8	1.1 ± 2.0	0.3 – 1.8	17.1 ± 13.3	12.2 – 22.1
Last dentist visit (missing = 5)						
<2 years ago (*n* = 14)	4.1 ± 6.6	0.3 – 7.9	1.4 ± 2.9	−0.3 – 3.1	13.1 ± 10.8	6.9 – 19.4
≥2 years ago (*n* = 40)	2.9 ± 4.0	1.7 – 4.3	1.4 ± 2.2	0.6 – 2.1	19.3 ± 13.9	14.9 – 23.8
Untreated caries (missing = 6)						
DT = 0 (*n* = 19)	-	-	-	-	17.2 ± 13.0	10.9 – 23.4
DT ≥1 (*n* = 34)	-	-	-	-	17.6 ± 13.7	12.8 – 22.4
Severe odontogenic consequences (missing = 9)						
PUFA = 0 (*n* = 25)	-	-	-	-	11.1 ± 7.2	8.1 – 14.1
PUFA ≥1 (*n* = 25)	-	-	-	-	21.8 ± 14.8*	15.6 – 27.9

All variables presented with standard deviations (s.d.) and 95% confidence intervals (95% CI).

s.d.: standard deviation; CI: confidence interval; -: not analysed; PUFA: Pulpal, Fistula, Ulceration, Abscess; OHIP: Oral Health Impact Profile questionnaire.

*Statistically significant, Mann-Whitney U test or Kruskal-Wallis test (*p* < 0.05); #: missing = 1; ##: missing = 4; ###: missing = 5.

## Discussion

The main findings of this study illustrate a high prevalence of untreated caries, severe odontogenic consequences and a poor OHRQoL in this population of marginalised Dutch adults. The assumption that this study population refrains from regular visits to the general dental practice is supported by the finding that the majority of participants last visited a dentist at least 2 years ago, and almost half did so more than 5 years ago. The assumed low SEP and vulnerability of the study population can be seen as corroborated by the high unemployment rate, a generally low level of education and high scores on the DiPCare-Q questionnaire.

The DiPCare-Q questionnaire is an instrument for measuring deprivation on an individual level, scored in three domains (social, material, health) and can be used to provide socioeconomic details about a population. When developing the DiPCare-Q, Vaucher et al. [27] used a highly heterogenic population of similar age, which they argue to be representative for many cultural backgrounds and most Western-European countries. Accordingly, we consider this questionnaire suitable for measuring the deprivation level of our study population. As the DiPCare-Q has scarcely been used or reported before, there are no reference values, and comparison with other populations or studies is precluded. Answers to individual DiPCare-Q items, however, portrayed the living conditions of the study participants on a material and social level more clearly. These findings not only indicate these individuals’ low SEP, but they also clarify the major socioeconomic deprivation and extreme poverty they experience. This information may be important for understanding the causes of oral health inequality and may inform the development of interventions in which the social context of the individual is considered.

The presence of untreated caries with pulpal involvement was associated with significantly poorer OHRQoL, whereas the presence of untreated caries alone was not related to a poorer OHRQoL. OHIP-14 items 3 and 4 concerning pain and discomfort were most prevalent in this population. Therefore, this may be explained by the fact that caries with an ICDAS score of three or higher can range from superficial caries to lesions involving the pulp. Generally, a higher chance of pain can be expected when caries is closer to the nerve, although this varies among individuals and depends on factors such as diet and the rate of caries progression [33–36]. This possibly explains why untreated caries alone does not result in poorer OHRQoL, as it can be superficial, whereas untreated caries with pulpal involvement most likely results in more pain and consequently a poorer OHRQoL can be anticipated. The mean OHIP-14 score of the population was 17.7 and was also not significantly different form OHIP-14 scores in the presence or absence of untreated caries (17.6; 17.2, respectively). The mean OHIP-14 score of our study population was significantly higher compared to the mean score of a representative Dutch adult population (2.8 ± 5.9) [37]. Our results suggest that other factors besides untreated caries, such as unfavourable social conditions, severe odontogenic consequences and other oral conditions may result in poorer OHRQoL, which is consistent with the literature [7, 37–40].

Considering the known association between low SEP and oral disease burden, unsurprisingly, our results showed a high prevalence of untreated caries in the study population [1, 4, 7, 12, 41]. While caries experience (DMFT) in the study population was similar to the general adult Dutch population of similar age (12.3 vs. approx. 12.7, respectively), participants experienced more untreated caries (DT) than the general adult Dutch population (3.3 vs. approx. 1.0, respectively) [23]. This may indicate an increased oral disease burden and need for professional oral healthcare which is now lacking. Our study additionally illustrates the severity of untreated caries as a consequence of dental care deprivation (i.e., caries that had reached the pulp [PUFA]). More than half of the study participants with untreated caries had lesions that had progressed into the pulp. The PUFA index was developed for documenting the consequences of untreated caries in children in developing countries [26, 41, 42]. This index has not yet been widely used among adults in high-income countries. To provide context to our findings: a study by Needleman et al. reported a PUFA prevalence of 8% in a population of professional UK football players aged 18–39 [43]. Public Health England reported a PUFA prevalence of 7% in adults aged 18 years or older, while a 33% prevalence of odontogenic infections (PUFA) was found amongst 311 individuals who had visited premises where unscheduled and out-of-hours treatment was provided [44].

When asked why individuals forgo routine oral healthcare, most reported to do this for financial reasons or because of fear for the dentist/dental treatment and/or shame for the dentition. This is in line with previous research, which reported that one in eight of the poorest 20% of the population forego oral healthcare for financial reasons, but only one in 100 of the richest 20% and one in 150 of the remaining 60% of the population tend to do so [45]. Some may forego general oral healthcare in any form for financial reasons. Others may visit a dental practice but find dental treatment too expensive and request ‘cheaper options’. A recent survey study by Broers et al. showed that 77% of patients who presented with an odontogenic infection at the dental practice chose extraction (i.e., the ‘cheaper option’) instead of conservation with endodontic treatment (i.e., the ‘expensive option’) [25]. This finding contributes to our understanding of the extent of the problem of affordability and accessibility of oral healthcare in a larger context, where socioeconomically vulnerable adults experience difficulties in receiving much needed care but adults in more favourable positions compromise on quality of care because of financial reasons.

Because the occurrence of missing values was associated with specific participant-related factors, we assume these to be missing completely at random (MCAR). Accordingly, we do not expect selection bias to be attributable to the use of a complete case analysis [46]. This study was not designed for causal comparison, nor do the findings allow for causal inference or generalisations about prevalence rates. The DipCare-Q questionnaire, which has been validated in English, was translated to Dutch without subsequent validation. This could have potentially affected the validity of the results. However, the questionnaire remained clear and concise (face validity), and most participants completed the questionnaire in the presence of the researcher, who provided clarification when needed. Given that the questionnaire addresses personal issues, such as drug and alcohol consumption, responses may have been influenced by social desirability bias. The three examiners who conducted the intraoral data collection were experienced dentists and instructed beforehand, but not calibrated. To ensure consistency, one researcher was present at all times to oversee the data collection and to provide a second opinion if needed. Given the clear clinical presentation of a PUFA score, the impact on the PUFA index is considered minor.

In interpreting our study’s findings, it should be considered that we included a selective population, which is unlikely to be seen in general dental practice and that the selection procedure may have introduced a degree of bias, warranting caution in interpreting the results. Participants were more likely to accept the invitation for free dental care if they experienced oral problems. Conversely, they may have been less likely to participate if they had a fear of the dentist or dental treatment, felt embarrassed about their dentition, or were unavailable on the treatment day. Because the treatment was administered at a mobile clinic that visited the participating organisation, it is estimated that minimal bias was introduced by participants’ immobility. While we found a negative effect of untreated caries with pulpal involvement on OHRQoL, other potentially influencing factors were not considered in this study. Given these considerations, our study population cannot be compared with findings from studies using random sampling from the general population or similar populations, nor with randomised studies on the effects of untreated caries. However, given the consistent results obtained across the included locations that were evenly distributed throughout the Netherlands, we anticipate similar outcomes among this vulnerable group within community organisations across the country. Considering the high disease burden in this ‘hard-to-reach’ population, the findings of this study remain significant in terms of reporting the consequences of untreated caries.

In conclusion, our findings on a marginalised adult population in the Netherlands demonstrate high prevalences for untreated caries and odontogenic infections with effects on the Oral Health-Related Quality of Life. This highlights the need for prompt changes in current oral healthcare policy and dental public health strategies to include these individuals in the professional oral healthcare system for both treatment and prevention of oral diseases. Additionally, it may emphasise the necessity of improving the socioeconomic circumstances of this population to prevent future oral diseases, as supported by the literature [1, 3–12]. These results are valuable for policymakers and oral healthcare professionals, as they highlight a population that is easily overlooked in their practices. For social workers in community organisations, this study could be important to create awareness of the likelihood that their clients will experience untreated oral health issues, adversely impacting their quality of life. The use of the DiPCare-Q and PUFA index in this atypical population uncovered contributing factors and consequences of poor oral health and neglected caries in this population. Future studies should explore the exact barriers and facilitators of marginalised and low SEP adults in their access to oral healthcare and explore the use of community organisations in accessing and engaging them for oral health purposes.

## Supplementary Material

Unequal smiles: consequences of untreated dental caries in citizens living in vulnerable circumstances in the Netherlands: an exploratory pilot study

Unequal smiles: consequences of untreated dental caries in citizens living in vulnerable circumstances in the Netherlands: an exploratory pilot study

## Data Availability

The data that support the findings of this study are available from the corresponding author, S. J. Gitz, upon reasonable request.
