# Supplementary material for: Unequal smiles: consequences of untreated dental caries in citizens living in vulnerable circumstances in the Netherlands: an exploratory pilot study
Source: Acta Odontol Scand. 2024 Oct 3;83:42028. doi: 10.2340/aos.v83.42028 (PMC11460080; doi:10.2340/aos.v83.42028)
Supplement: Unequal smiles: consequences of untreated dental caries in citizens living in vulnerable circumstances in the Netherlands: an exploratory pilot study [file AOS-83-42028-s1.pdf]

Supplementary material has been published as submitted. It has not been copyedited or typeset by Acta Odontologica Scandinavica.

Supplementary Material

Variable recoding

Tooth brushing frequency and date of last visit to a dentist were dichotomised into: toothbrushing ≥ 2 or < 2 times a day and last dentist visit ≥ 2 or < 2 years ago. Level of education was stratified according to the CBS 2019 grouping (low, middle, high) (48). The reasons for forgone professional oral healthcare were categorised into: financial reasons, fear of the dentist/dental treatment and/or embarrassment of dentition, fear of having undocumented status discovered, not needing professional oral healthcare, personal circumstances, difficult access to professional oral healthcare, and no reason. Some participants gave multiple answers, which were counted in multiple categories. This resulted in a total prevalence that exceeds 100%. As per author instructions, the DiPCare-Q dimensions material deprivation (0–8 points) and health deprivation (0–3 points) were scaled into three and two levels, respectively. Items in the social deprivation dimension were recoded so no-answers were equal to one point, whereas yes-answers in the material and health dimensions were equal to one point. Recoded data were used for analysing and calculating the overall DiPCare-Q index (27).
