# Supplementary material for: Unequal smiles: consequences of untreated dental caries in citizens living in vulnerable circumstances in the Netherlands: an exploratory pilot study
Source: Acta Odontol Scand. 2024 Oct 3;83:42028. doi: 10.2340/aos.v83.42028 (PMC11460080; doi:10.2340/aos.v83.42028)
Supplement: Unequal smiles: consequences of untreated dental caries in citizens living in vulnerable circumstances in the Netherlands: an exploratory pilot study [file AOS-83-42028-s2.pdf]

Supplementary material has been published as submitted. It has not been copyedited or typeset by Acta Odontologica Scandinavica.

Table S1. Prevalence of sociodemographic details of the study population (N=59) and the general Dutch population (23,47).

**Variable**	**Study population n (%)**	**General population (%)**
Demographics[23]		
Low education level (missing = 7)	22 (42.3)	25.0
Immigration background (missing = 1)	40 (69.0)	24.6
Unemployment (missing = 5)	47 (87.0)	3.8
Oral hygiene habits[47]		
Last dentist visit ≥ 2 years ago (missing = 4)	40 (74.5)	11.5
Brushing ≤ 1 times a day (missing = 4)	24 (43.6)	27.9

Table S2. Prevalence of responses** per dimension and per item (1 to 16) and median score (IQR) per dimension of the DiPCare-Q questionnaire in the study population (n = 54)

**Dimension**	**Median (IQR)**	**Yes response frequency n (%)****
Material (0 to 8 points)	5 (3 to 7)	51 (94.4)
2. Need to borrow money for daily expenses		37 (68.5)
5. Cannot afford clothes for self/family members		35 (64.8)
1. Difficulty paying household bills		34 (63.0)
13. Difficulty paying back loans		34 (63.0)
3. Forgoing healthcare for family members		31 (57.4)
6. Cannot afford household items for self/family members		27 (50.0)
4. Fear of losing housing		26 (48.1)
10. Not enough to eat at home		24 (44.4)
Health (0 to 3 points)	1 (0 to 2)	32 (59.3)
15. Mental health issues		25 (46.3)
14. Physical disability		14 (25.9)
16. Drug, alcohol or gambling addictions		10 (18.5)
		**No response frequency n (%)****
Social (0 to 5 points)	3 (2 to 3)	54 (100)
7. Holidays		50 (92.6)
9. Leisure/recreational activities		50 (92.6)
8. Evenings spent with family or friends		19 (35.2)
12. Person to turn to for material support		18 (33.3)
11. Access to the internet		13 (24.1)

Abbreviations: IQR, interquartile range; ** a yes response (1 point) in the material and health dimensions equals deprivation, whereas in the social dimension a no response (1 point) equals deprivation.
